# Metabolic responses to a 48-h ultra-marathon run in middle-aged male amateur runners

**DOI:** 10.1007/s00421-013-2714-8

**Published:** 2013-09-04

**Authors:** Barbara Kłapcińska, Zbigniew Waśkiewicz, Stanisław J. Chrapusta, Ewa Sadowska-Krępa, Miłosz Czuba, Józef Langfort

**Affiliations:** 1 Department of Physiological and Medical Sciences, The Jerzy Kukuczka Academy of Physical Education, 72A Mikołowska St, 40-065 Katowice, Poland; 2 Department of Experimental Pharmacology, Mossakowski Medical Research Center Polish Academy of Sciences, Warsaw, Poland

**Keywords:** Ultra-marathon, Muscle damage, Inflammation, Interleukin-6, Cardiac biomarkers, Acid–base balance

## Abstract

**Purpose:**

To evaluate ongoing metabolic changes during a 48-h competitive run and a 48-h recovery period, with focus on potential health risks exemplified by heart and skeletal muscle damage biomarkers and oxidative stress-related indices.

**Methods:**

Blood samples were taken before the race, after 12, 24, and 48 h of running, and after 24 and 48 h of recovery from male amateur runners (*N* = 7, age 35–59 years, VO_2max_ mean ± SD 57.0 ± 4.0 ml kg^−1^ min^−1^, total distance covered 183–320 km). The samples were analyzed for morphology, acid–base and electrolyte balance, iron status, lipid profile, interleukin-6, high-sensitivity C-reactive protein, N-terminal pro-brain-type natriuretic peptide, high-sensitivity cardiac troponin T, non-enzymatic antioxidants, activities of selected enzymes including antioxidant enzymes, and total antioxidant status.

**Results:**

The sustained ultra-endurance run caused hypocapnic alkalosis with slight hyperkalemia and hypocalcemia, but no hyponatremia. Blood biochemistry showed severe muscle but not liver damage, and an acute inflammatory response. These effects were evidenced by leukocytosis, several fold rises in interleukin-6 and high sensitivity C-reactive protein, extreme elevations in serum levels of muscle enzymes, and marked increases in cardiac biomarker levels. Most of the changes dissolved during the 48 h post-race recovery. Neither the iron pool, nor erythropoiesis, nor pro-oxidant/antioxidant balance were substantially affected.

**Conclusions:**

The changes consequent on the ultra-endurance run do not pose a serious health risk in men who begin their endeavor with ultra-endurance running in mid-life. There is some circumstantial evidence that hyperventilatory hypocapnia may modulate inflammatory response by stimulating the release of interleukin-6 from working skeletal muscles.

## Introduction

It is well established that regular physical activity is beneficial for cardiovascular health (Shiroma and Lee 2010) as well as for adult neurogenesis and cognitive performance (Lafenetre et al. 2011). It also helps preventing lifestyle-related metabolic diseases (Sato et al. 2003) as well as some cancers (World Cancer Research Fund 2007), and improves survival and quality of life of cancer survivors (Speck et al. 2010; West-Wright et al. 2009). Owing largely to these and related findings, jogging and low-intensity running has become a fast-growing sporting activity practiced all over the world and involving growing numbers of participants of both genders and broad age range. As with most new ideas that are put into practice, this one has also been taken to an extreme by some of its adherents. As a result, some of those who initially engaged in this activity as a health-promoting or recreational measure moved toward more intense and longer duration running events, including the extreme form which is marathon and ultra-marathon races.

There is relatively little data on the health-related effects of ultra-endurance runs. However, a number of studies revealed that repeated prolonged strenuous exercise, such as ultra-long distance running, may be hazardous to athletes health, as manifested by large-artery wall stiffening and abnormalities in left and right ventricular function (for review see O’Keefe et al. 2012), atrial fibrillation (Mascia et al. 2013), excessive muscle and cartilage damage (Kim et al. 2009; Bessa et al. 2008; Suzuki et al. 2006), and systemic inflammatory reaction (Toumi et al. 2006; Wallberg et al. 2011; Butterfield et al. 2006; Tidball 2005). Some people involved in amateur long-distance and/or ultra-long distance running start exercising at middle age, after a long period of sedentary lifestyle; the consequences of this change in lifestyle remain mostly unexplored. The main objective of this study was to evaluate, in this specific athletes subpopulation, acute changes in a wide spectrum of hematological and biochemical indices, which are evoked by running distances extending far beyond that of the classic marathon, namely by a 48-h ultra-marathon run, with particular consideration of potential health risks. For this purpose, we employed the so-called sportomics strategy, which represents a holistic top–down approach (also known as ex post facto design) (see Resende et al. 2011; Gonçalves et al. 2012). This investigation, which was designed with consideration of the importance of in-field metabolic analyses (Resende et al. 2011), is a sequel to our earlier study performed in a similar group of ultra-endurance runners who competed in a 24-h ultramarathon run (Waśkiewicz et al. 2010), but additionally includes data on some biomarkers of cardiac stress that were not explored previously.

## Methods

### Participants

The study group consisted of seven men (out of the total of 44 participants of the ultra-marathon) who declared to provide blood samples during and after the 48-h ultra-marathon race organized by the Academy of Physical Education in Katowice, Poland. Before the start of the run, all the runners were assessed for body composition using a model In Body220 (Biospace Inc., Seoul, Korea) analyzer, completed a survey on their medical and training history, and signed an informed consent to participate in the study after being informed about the experimental procedures and risks involved. The study protocol was designed in accordance with the Declaration of Helsinki and was approved by the Research Ethics Committee of the Academy.

### Study protocol

The runners competed on a 3,000 m oval walking trail located in a perfectly flat area in Katowice outskirts. Weather conditions at the start of the race (12 p.m.) were good (22 ºC and 53 % relative humidity), but they worsened thereafter and reached 12 ºC and 94 % relative humidity at the finish of the race. The runners were allowed free (unrecorded) intake of food (sandwiches, cookies, pasta, bananas, and carbohydrate energy bars) and liquids (water, sport beverages, soft drinks, and broth). Each participant received an electronic chip to allow automated counting of the passages through the electronic gate set up on the trail to estimate the total distance covered. At the run finish, the distance from the last passage through the gate was measured manually. The runners were allowed to take rest (including sleep) at their free will during the run. The duration of such breaks was recorded and taken into account when calculating the actual running speed, whereas the duration of short breaks (e.g., those for snacking, etc.) was not. Samples of venous and fingertip capillary blood were taken 3 h before the start of the competition, after 12 and 24 h of running, within 10 min after completion of the run, and then after 24 and 48 h of recovery. Within 1 h of blood taking, the samples were carried to a nearby clinical laboratory for processing and analyses.

Two weeks after the run, the participants were subjected to physiological testing to assess their maximal oxygen uptake (VO_2max_) and the lactate threshold (LT). As ultra-endurance runners mostly rely on high volume training at low to moderate intensities, with no speed training targeted at the development of motor coordination required for fast running, the test consisted of a combination of varying treadmill speed and grade. The test was performed on a model LE 200 treadmill (Jaeger, Germany), beginning at 6 km/h and 0° inclination. Treadmill speed was increased by 2 km/h every 3 min until reaching 14 km/h. Then, keeping the speed steady, the grade was increased by 2.5 % every 3 min [to ensure similar increments in the workload (WR) on subsequent steps of the test, see Porszasz et al. 2003] and the run continued until volitional fatigue. If the last step of the test was not completed, the maximal workload was calculated using the following formula:

$${\text{WRmax}} = {\text{WR}}_{\text{f}} + \left( {{\text{t}}/{\text{T}} {\star} {\text {WR}}_{\text{i}} } \right),$$

where “WR_f_” is the last completed workload in watts, “t” is the time in seconds of the incomplete step, “T” is the duration of each step, and “WR_i_” is the step-increase in WR in watts (Kuipers et al. 1985). During the test, heart rate, minute ventilation (VE), oxygen uptake (VO_2_), and expired carbon dioxide (CO_2_) were continuously measured using a model MetaLyzer 3B-2R stationary spiroergometer (Cortex, Leipzig, Germany) in the breath-by-breath mode. Fingertip capillary blood samples for the assessment of lactate (LA) concentration were drawn at rest and at the end of each incremental step; using these data, individual LT was estimated by the D-max method (Cheng et al. 1992). VO_2max_ was determined based on the following criteria: (1) a plateau in VO_2_ at rising WR (ΔVO_2_ ≤ 150 mL/min at VO_2_ peak), (2) maximal respiratory exchange ratio ≥1.1, and (3) LA concentration ≥8 mM. All gas exchange data were time-averaged using 15 s intervals to examine the VO_2_ plateau.

Anthropometric and physiological characteristics of the participants are presented in Table 1. Considering the essential indices of performance in marathon running, namely the anaerobic threshold, fractional utilization of maximal oxygen uptake (%VO_2max_), and running economy (Millet et al. 2011), one may note that LT values in these athletes matched those of elite, world-class endurance athletes, whereas their mean value of VO_2max_ was lower than that recorded in champion endurance athletes (Joyner and Coyle 2008), but was comparable to those for endurance-trained runners (Wilmore and Costill 2005).

**Table 1:** Anthropometric and performance characteristics of participants

Variable	Mean ± SD	Min–Max
Age [years]	45.4 ± 9.2	35–59
Training history [years]	5 ± 2	3–8
Body mass index [kg/m^2^]	22.0 ± 1.6	20.4–22.2
Body fat [%]	10.5 ± 4.8	4.7–18.3
Weekly covered distance [km]	83 ± 32	30–115
VO_2max_ [ml O_2_/kg/min]	57.0 ± 4.0	53.0–65.0
Individual anaerobic threshold [ml O_2_/kg/min]	48.3 ± 3.9	44.0–56.0
LT [%VO_2max_]	84.3 ± 3.2	80.0–90.0
Velocity at LT [km/h]	13.6 ± 1.0	12.0–14.0
Workload at LT [W/kg]	3.6 ± 0.3	3.2–4.01
Maximal workload [W/kg]	4.7 ± 0.4	4.2–5.3
VE max [l/min]	155 ± 30	121–194
Peak heart rate [bpm]	183 ± 12	168–201
Peak serum lactate [mmol/l]	9.3 ± 2.7	7.4–15.0

### Biochemical analyses

Blood gases, electrolytes, LA, and glucose in fingertip capillary blood were determined on a model GEM Premier 3000 analyzer (Instrumentation Laboratory). Blood morphology was assessed in EDTA-anticoagulated venous blood on an automated hematology analyzer XS-1000i (Sysmex). Serum ferritin was measured with an Abbott Architect i2000 SR analyzer, serum Fe—on an Olympus AU400 analyzer (Olympus, Japan) and plasma soluble transferrin receptor (sTfR) level—on a BN-ProSpec nephelometer (Dade-Behring-Siemens). Serum activities of creatine kinase (CK; EC.2.7.3.2), γ-glutamyl transferase (GGT; EC.2.3.2.2), aspartate aminotransferase (AST; EC.2.6.1.1), alanine aminotransferase (ALT; EC.2.6.1.2) and lactate dehydrogenase (LDH; EC.1.1.1.27), and serum total cholesterol (TC), HDL-cholesterol (HDL-C) and triglycerides (TG) were measured on a model Synchron CX 9 Pro (Beckman-Coulter) analyzer. Samples with activities exceeding the upper reference limits were rerun with Overrange Detection and Correction (ORDAC) enabled, or were diluted with saline and reanalzyed, the dilution factor was entered into the sample information. The intra-assay coefficients of variation (CV) for these assays were 5.8, 5.6, 6.7, 5.3, and 5.6 %, and 3.5, 6.1, and 4.5 %, respectively. Serum LDL-cholesterol (LDL-C) was calculated by the Friedewald formula. Serum free fatty acids (FFA), glycerol, and β-hydroxybutyrate (βHB) were assessed using manual test kits NEFA, Glycerol and Ranbut (CV 2.7, 1.3, 4.7 %, respectively) (Randox Laboratories, Antrim, UK). Serum high-sensitivity C-reactive protein (hsCRP) was assessed using a Dade-Behring (Marburg, Germany) kit (intra-assay CV 3.5 %), and total serum interleukin 6 (IL-6) was determined using Human High Sensitivity ELISA kit (Gen-Probe Diaclone SAS, France; CV: 4.4 %). Blood activity of superoxide dismutase (SOD, EC 1.15.1.1) and glutathione peroxidase (GPX, EC 1.11.1.9) were determined in red blood cells (RBC) lysates using Randox diagnostic kits (Ransod SD125 and Ransel RS504, respectively), the activity of catalase (CAT, EC 1.11.1.6) was measured by the method of Aebi (1984), and that of glutathione reductase (GR, EC 1.6.4.2)—by the method of Glatzle et al. (1970). Reduced glutathione (GSH) was assessed by a routine colorimetric method with Ellman’s reagent. Thiobarbituric acid-reactive substances (TBARS) were determined using *n*-butanol extraction (Buege and Aust 1978) and expressed as nmol of malondialdehyde/L. Plasma total antioxidant status (TAS) was quantified with the Randox TAS kit NX2332 and expressed as mmol Trolox equivalent/L. Plasma concentration of uric acid was assessed with the Randox UA 230 kit.

Serum samples meant for cardiac marker assays were frozen and stored at −80 °C until analyzed. N-terminal pro-brain-type natriuretic peptide (NTproBNP) and high-sensitivity cardiac troponin T (hs-cTnT) were assayed with a model Cobas E601 analyzer (Roche Diagnostics) using electrochemiluminescence method.

### Statistics

Data are presented as mean ± SD. All data were tested for homogeneity of variances using the Brown–Forsythe test and for distribution normality by Shapiro–Wilk’s test, and the data showing significant heterogeneity of variances and/or major deviations from normality (skewness and/or kurtosis outside the [−4.0, +4.0] interval) were normalized by log_10_ transformation prior to further analysis. Next, the data were analyzed by one-way repeated measures ANOVA followed by Student‘s *t* test for dependent variables, with sequential Bonferroni–Holm correction. Comparisons between younger and older runner subsets were performed by the Mann–Whitney *U* test because of low sample sizes. Spearman’s rank correlation coefficient (*R*) was calculated to assess associations between variables; differences between *R* values were tested by a single-sided test. In all cases, a *p* < 0.05 was considered significant. All statistical analyses were run using Statistica 7.1 software package (StatSoft, Tulsa, OK, USA).

## Results

### Ultra-marathon performance (see Table 2)

Actual running speed, calculated based on the time actually spent on the track (i.e. after subtracting rest/sleep breaks) during the respective 12 h time intervals, reached a minimum in the 1st half of the 2nd day of the race. The younger runners (<40 years of age, *N* = 3, mean ± SD 37.0 ± 1.6 years) compared to the older ones (*N* = 4, mean age 51.8 ± 5.7 years) covered but nonsignificantly longer distances (296.5 ± 27.3 km vs. 267.3 ± 51.3 km, *p* = 0.85 by the Mann–Whitney test). Similar tendencies, in favor of the younger athletes, were found in laboratory measures of endurance capacity (VO_2max_, individual anaerobic threshold, LT, V_E max_, peak serum lactate, and velocity at LT (data not shown; *p* ≥ 0.11 by the Mann–Whitney test). None of the participants experienced an adverse medical event during or after the run.

**Table 2:** Ultra-marathon race performance measures

	0–12 h running		13–24 h running		25–36 h running		37–48 h running	
	Total distance covered [km]	Actual running speed [km/h]	Total distance covered [km]	Actual running speed [km/h]	Total distance covered [km]	Actual running speed [km/h]	Total distance covered [km]	Actual running speed [km/h]
Mean ± SD	102.6 ± 8.3	8.6 ± 0.7	169.3 ± 18.7	6.0 ± 0.6***	222.9 ± 34.9	4.7 ± 1.1***^#^	279.8 ± 48.7	6.3 ± 0.9**
Median	102.0	8.5	174.0	6.0	228.0	4.9	290.6	5.9
Min–Max	88.5–114.0	7.4–9.5	133.5–192.0	5.0–6.8	156.0–259.5	3.2–5.9	182.7–319.7	5.2–8.1

Actual running speed ANOVA results: *F*_3,18_ = 24.8, *p* = 10^-5^; ** *p* < 0.01, *** *p* < 0.001 versus the running speed for the first 12 h; ^#^ *p* < 0.05 versus the running speed for the preceding 12 h; Student *t* test for dependent variables, with the sequential Bonferroni–Holm correction

### Blood acid–base balance, electrolytes, and iron status (see Table 3)

Pre-race levels of capillary blood gases and acid–base balance indices were within normal ranges. Sustained running-related hyperventilation led to significant declines in mean pCO_2_ and mean pCO_2_/HCO_3_^−^ ratio (from 1.643 ± 0.036 to 1.408 ± 0.103), and to a significant increase in mean blood pH that was apparent throughout the entire observation period. During the run, both pCO_2_ and pH were significantly correlated with distance covered (*R* = −0.58, *p* < 0.01, and *R* = 0.79, *p* < 0.001, respectively). Glucose level showed no considerable change during the entire observation period, while LA level, which did not change significantly during the race, was significantly lower 24 h post-run. Blood sodium decreased progressively during the run, but it dropped transiently below the normal range only in two runners. Hyperkalemia that developed in a growing number of participants over the course of the race disappeared in all of them after 24 h recovery. Blood [K^+^] correlated positively with distance covered (*R* = 0.52, *p* < 0.01) and with actual running speed (*R* = 0.55, *p* < 0.05). A progressive decrease in mean blood Ca^2+^ level was observed during the run, which correlated with total distance covered (*R* = −0.47, *p* < 0.05), but only one runner experienced a borderline hypocalcemia ([Ca^2+^] = 1.12 mmol/L) at the end of the competition, which dissolved during the next 24 h.

**Table 3:** Ultra-marathon-induced changes in blood acid base balance, iron status and levels of electrolytes, glucose and lactate

Variable	Reference range	Pre-race	12 h running	24 h running	48 h running	24 h post-race	48 h post-race	ANOVA results	
pH	7.350–7.450	7.394 ± 0.011	7.428 ± 0.026*	7.440 ± 0.016**	7.462 ± 0.023***	7.440 ± 0.016***	ND	**F**_**4,24**_ **=** **13.0**	***p*** **<** **10** ^**−3**^
pCO_2_ [mmHg]	35–45	43 ± 2	38 ± 4.5**	38 ± 2**	36 ± 2***	41 ± 1**	ND	**F**_**4,24**_ **=** **15.3**	***p*** **<** **10** ^**−3**^
Ca^2+^[mmol/l]	1.13–1.30	1.22 ± 0.03	1.19 ± 0.07	1.18 ± 0.05*	1.15 ± 0.02***	1.17 ± 0.02*	ND	**F**_**4,24**_ **=** **10.2**	***p*** **=** **0.005**
Na^+^[mmol/l]	135–145	141 ± 2	140 ± 2	138 ± 3	137 ± 3*	140 ± 4	138 ± 1**	**F**_**5,30**_ **=** **3.76**	***p*** **=** **0.009**
K^+^ [mmol/l]	3.5–5.1	4.6 ± 0.2	5.1 ± 0.6	5.3 ± 0.8	5.3 ± 0.6	4.7 ± 0.4	4.6 ± 0.4	**F**_**5,30**_ **=** **3.84**	***p*** **=** **0.008**
Glucose [mmol/l]	3.9–5.8	5.3 ± 0.9	4.9 ± 1.2	5.9 ± 0.9	6.1 ± 0.8	5.4 ± 0.6	5.0 ± 0.7	F_5,30_ = 2.47	*p* = 0.055
Lactate [mmol/l]	0.5–2.4	2.4 ± 0.5	2.3 ± 0.5	2.1 ± 0.5	2.0 ± 0.7	1.6 ± 0.4***	ND	**F**_**4,24**_ **=** **3.50**	***p*** **=** **0.022**
Fe [μmol/l]	10.0–30.8	15.0 ± 5.2	8.1 ± 5.3	14.5 ± 4.8	5.9 ± 1.7*	12.5 ± 8.4	ND	**F**_**4,24**_ **=** **3.80**	***p*** **=** **0.016**
Ferritin [μg/l]	22–275	49 ± 30	64 ± 39*	81 ± 47**	136 ± 66**	125 ± 45***	ND	**F**_**4,24**_ **=** **22.6**	***p*** **<** **10** ^**−3**^
sTfR [mg/l]a	0.83–1.76	1.13 ± 1.02	1.11 ± 0.96	1.01 ± 0.84*	0.95 ± 0.79***	0.86 ± 0.68***	ND	**F**_**4,24**_ **=** **34.7**	***p*** **<** **10** ^**−3**^
sTfR/log_10_Ferritin index		0.72 ± 0.15	0.66 ± 0.14***	0.56 ± 0.13***	0.46 ± 0.11***	0.42 ± 0.08**	ND	**F**_**4.24**_ **=** **58.2**	***p*** **<** **10** ^**−3**^

*sTfR* soluble transferrin receptor, * *p* < 0.05, ** *p* < 0.01, *** *p* < 0.001 versus the respective pre-race value; the significance levels shown with asterisks refer to *p* values with the sequential Bonferroni–Holm correction. *ND* not determined, statistically significant ANOVA results are shown in boldtype

Serum Fe level showed highly dissimilar individual patterns of changes during the run (not shown). Two runners were slightly hypoferremic already at the start of the race, but serum Fe at the end of the run was below the lower reference limit in all participants and remained low (≤7 μmol/l) in three of them 24 h later. Serum ferritin was within normal range in all runners at the beginning of the race, showed a progressive and marked (two- to fivefold) elevation in all runners during the run and remained elevated 24 h post-race, but in no case crossed the upper limit of the normal range. It correlated moderately (*R* = −0.44, *p* < 0.01) with serum Fe, and strongly (*R* > 0.71, *p* < 0.001) with serum hsCRP and muscle damage markers (CK, LDH, AST, ALT). Mean serum sTfR level and sTfR/log_10_Fer index declined steadily for the observation period. The latter index correlated positively with serum Fe, Hb, RBC count, and Hct (*R* = 0.38, *R* = 0.37, *R* = 0.52, and *R* = 0.47, respectively, *p* < 0.05 for all) and negatively with serum CK activity and hsCRP level (*R* = −0.55 and *R* = −0.69, respectively, *p* < 0.001 for both).

### Blood morphology (see Table 4)

No significant change was found in mean hemoglobin (Hb) level and mean RBC count during the run, whereas mean hematocrit (Hct) showed a significant decrease at the end of the run. All the indices were significantly decreased during the post-race recovery, but remained within their normal ranges for males. MCV, MCHC, and MCH remained fairly stable for most of the study period. Mean platelet (PLT) count showed a significant but moderate and transient increase during the 1 day of the race, but platelet counts remained within normal range throughout the entire observation period in all participants. Mean total leukocyte (WBC) count peaked after 12 h of running, mainly due to a marked rise in neutrophil count, then decreased slightly over the next 36 h of the run, and returned to normal range 24 h post-race. Mean monocyte and lymphocyte counts peaked after the first 24 h, then showed a continuous decline for the remainder of the observation period.

**Table 4:** Ultra-marathon-induced changes in basic blood counts and selected circulating leukocyte subpopulations

Variable	Reference range	Pre-race	12 h running	24 h running	48 h running	24 h post-race	48 h post-race	ANOVA results	
Hb [g/dl]	11.2–15.8	13.9 ± 0.9	14.2 ± 0.6	14.0 ± 0.7	14.0 ± 1.0	12.2 ± 1.1**	12.2 ± 1.3**	**F**_**5,30**_ **=** **19.8**	***p*** **<** **10** ^**−3**^
RBC [×10^6^/μl]	4.0–5.8	4.7 ± 0.4	4.8 ± 0.3	4.7 ± 0.3	4.5 ± 0.4	4.1 ± 0.5**	4.1 ± 0.5**	**F**_**5,30**_ **=** **22.6**	***p*** **<** **10** ^**−3**^
HCT [%]	35–45	41 ± 3	41 ± 2	40 ± 2	38 ± 3**	36 ± 3**	36 ± 4**	**F**_**5,30**_ **=** **15.5**	***p*** **<** **10** ^**−3**^
PLT [×10^3^/μl]	130–400	238 ± 53	283 ± 74*	276 ± 66**	261 ± 55	219 ± 45	213 ± 46	**F**_**5,30**_ **=** **15.6**	***p*** **<** **10** ^**−3**^
MCV [fl]	82.0–98.0	87.7 ± 3.5	85.4 ± 3.2**	84.7 ± 3.0***	85.4 ± 2.9**	88.1 ± 3.7	89.3 ± 4.0*	**F**_**5,30**_ **=** **48.5**	***p*** **<** **10** ^**−3**^
MCH [pg]	27.0–30.0	29.7 ± 1.3	29.8 ± 1.2	29.8 ± 1.2	29.8 ± 1.2	30.0 ± 1.3	30.1 ± 1.3*	**F**_**5,30**_ **=** **5.40**	***p*** **=** **0.0012**
MCHC [%]	34.0–36.0	33.9 ± 1.0	34.9 ± 0.8**	35.1 ± 0.9***	34.9 ± 0.7*	34.0 ± 1.0	33.8 ± 1.1	**F**_**5,30**_ **=** **30.2**	***p*** **<** **10** ^**−3**^
WBC [×10^9^/l]	4.0–10.0	5.4 ± 0.8	14.9 ± 3.5**	13.5 ± 2.6***	12.9 ± 3.0**	7.1 ± 2.0	6.0 ± 1.5	**F**_**5,30**_ **=** **49.5**	***p*** **<** **10** ^**−3**^
Neutro*p*hils [× 10^9^/l]	1.8–7.7	3.2 ± 0.5	11.3 ± 3.5**	9.8 ± 2.2**	9.9 ± 3.3**	4.6 ± 2.4	3.9 ± 1.8	**F**_**5,30**_ **=** **38.0**	***p*** **<** **10** ^**−3**^
Monocytes [×10^9^/l]	0–0.8	0.5 ± 0.1	1.4 ± 0.3***	1.5 ± 0.5**	1.1 ± 0.4**	0.7 ± 0.2**	0.6 ± 0.2**	**F**_**5,30**_ **=** **37.5**	***p*** **<** **10** ^**−3**^
Lym*p*hocytes [×10^9^/l]	1.0–4.5	1.6 ± 0.5	2.0 ± 0.8	2.1 ± 0.9	1.8 ± 0.6	1.6 ± 0.5	1.4 ± 0.5	**F**_**5,30**_ **=** **4.82**	***p*** **=** **0.0023**

* *p* < 0.05, ** *p* < 0.01, *** *p* < 0.001 versus the respective pre-race value; the significance levels shown with asterisks refer to *p* values with the sequential Bonferroni–Holm correction. Statistically significant ANOVA results are shown in boldtype

### Blood biochemistry

Mean activities of CK, ALT, AST, and LDH increased progressively to reach, at the race finish, values 107-, 25-, 7-, and 5-fold higher, respectively, than their pre-race values, and greatly declined thereafter, but stayed markedly elevated 48 h post-race. Activities of all the enzymes during the run highly correlated with distance covered (*R* ≥ 0.86, *p* < 0.001) and showed large inter-individual variability at the end of the race (CV 105, 110, 98, and 90 %, respectively) and 48 h post-race (CV 85, 110, 86, and 84 %, respectively). Mean serum GGT activity showed a slight while significant decline during the run, which persisted 24 h post-race, but lost significance 24 h later (Table 5); GGT activity showed also relatively small (CV 19–32 %) inter-individual variability during the observation period.

**Table 5:** Ultra-marathon-induced changes in serum markers of skeletal or cardiac muscle damage and inflammation

Variable	URL	Pre-race	12 h running	24 h running	48 h running	24 h post-race	48 h post-race	ANOVA results	
CK [U/l]^a^	174	193 ± 79	5056 ± 4651***	18010 ± 18711***	20605 ± 21595***	6277 ± 6027***	1582 ± 1349***	**F**_**5,30**_ **=** **68.3**	***p*** **<** **10** ^**−3**^
AST [U/l]^a^	42	33 ± 6	138 ± 95**	541 ± 517**	813 ± 896**	401 ± 428**	228 ± 251*	**F**_**5,30**_ **=** **45.4**	***p*** **<** **10** ^**−3**^
ALT [U/l]^a^	40	30 ± 8	48 ± 20**	124 ± 95**	226 ± 220**	172 ± 150**	140 ± 121**	**F**_**5,30**_ **=** **26.2**	***p*** **<** **10** ^**−3**^
LDH [U/l]^a^	180	193 ± 38	377 ± 136**	700 ± 498**	1014 ± 916**	696 ± 569*	582 ± 487*	**F**_**5,30**_ **=** **23.6**	***p*** **<** **10** ^**−3**^
GGT [U/l]^a^	37	24 ± 8	ND	22 ± 8	21 ± 6*	20 ± 5**	21 ± 4	**F**_**4,24**_ **=** **4.09**	***p*** **=** **0.012**
NTproBNP [pg/ml]^a^	125	54 ± 50	299 ± 194**	508 ± 458**	329 ± 412**	ND	108 ± 86*	**F**_**4,24**_ **=** **21.7**	***p*** **<** **10** ^**−3**^
hs-cTnT [ng/ml]^a^	0.016	0.004 ± 0.002	0.014 ± 0.012*	0.008 ± 0.006	0.008 ± 0.008	ND	0.004 ± 0.002	**F**_**4,24**_ **=** **7.27**	***p*** **<** **10** ^**−3**^
IL-6 [pg/ml]^a^	4.72	0.64 ± 0.34	35.86 ± 17.35***	33.25 ± 16.54***	23.20 ± 18.85***	7.39 ± 13.32**	2.19 ± 3.67	**F**_**5,30**_ **=** **84.7**	***p*** **<** **10** ^**−3**^
CRP [mg/l]^a^	5.0	0.8 ± 0.8	3.4 ± 1.7**	30.0 ± 8.9***	63.5 ± 31.5***	45.5 ± 37.8***	28.0 ± 31.2**	**F**_**5,30**_ **=** **59.3**	***p*** **<** **10** ^**−3**^

Statistically significant ANOVA results are shown in boldtype

*ALT* alanine aminotransferase, *AST* aspartate aminotransferase, *CK* creatinine kinase, *CRP* C-reactive protein, *GGT* γ-glutamyltransferase, *hs-cTnT* high-sensitivity cardiac troponin T, *IL-6* interlekin 6, *LDH* lactate dehydrogenase, *NTproBNP* N-terminal pro-brain-type natriuretic peptide, *URL* upper reference limit, *ND* not determined

* *p* < 0.05, ** *p* < 0.01, *** *p* < 0.001 versus the respective pre-race value; the significance levels shown with asterisks refer to *p* values with the sequential Bonferroni–Holm correction

^a^ The ANOVA results and significance levels pertain to log_10_-transformed data

Marked running-related increases were also found in serum cardiac and inflammatory markers (Table 5). Mean levels of NTproBNP and hs-cTnT peaked during the first 12 and 24 h, respectively, and decreased thereafter, but only the latter normalized at the end of the observation period. NTproBNP level correlated moderately with hs-cTnT level (*R* = 0.52, *p* < 0.01), and they both correlated moderately with actual running speed (*R* = 0.55 and *R* = 0.53, respectively, *p* < 0.01). Mean serum IL-6 level peaked two orders of magnitude above the pre-race value after the first 12 h, then showed some decrease toward the end of the competition and normalized 48 h post-race; changes in mean serum IL-6 level roughly followed those in hs-cTnT (*R* = 0.49; *p* < 0.01). Mean hsCRP level rose continuously throughout the run to a maximum 75-fold higher than baseline, then showed a marked decrease during the recovery, but still remained significantly elevated 48 h post-race (range 8.7–98.0 mg/l). IL-6 and hsCRP levels showed, respectively, moderate and very strong correlation with distance covered (*R* = 0.43, *p* = 0.02 and *R* = 0.93, *p* < 10^−3^, respectively). IL-6 level also strongly correlated with actual running speed (*R* = 0.75, *p* < 10^−3^), whereas hsCRP level did not (*R* = 0.28, *p* = 0.15).

The most marked changes in lipid metabolism-related indices (Table 6) were the decrease in circulating TG level that stabilized at about 60 % of the pre-race value after the first 24 h of running and increases in circulating FFA (>sixfold), glycerol (11-fold), and βHB (threefold) that reached their respective maxima after the first 12 h; all these changes dissolved 48 h post-race. As expected, βHB level correlated strongly with FFA and glycerol levels (*R* = 0.73 and *R* = 0.76, respectively, *p* < 0.001). Changes in the other lipid profile measures during the run were progressive and unidirectional. Circulating LDL-C and TC levels declined relatively little, but remained decreased 48 h post-race, while HDL-C level rose progressively for the entire duration of the run and returned to baseline 48 h post-race (Table 6). Significant declines in TC/HDL-C, LDL-C/HDL-C, and TG/HDL-C ratios and in AIP - Atherogenic Index of Plasma (Dobiášová and Frohlich 2001) were found during the run, while opposite trends were evident during the recovery. During the run, serum TG, TC, and LDL-C levels, all the lipid ratios and AIP correlated negatively (−0.87 < *R* < −0.52, *p* < 0.01), while serum HDL-C level correlated positively (*R* = 0.66, *p* < 0.01) with the distance covered.

**Table 6:** Ultra-marathon-induced changes in serum lipid profile

Variable	Reference range/URL	Pre-race	12 h running	24 h running	48 h running	24 h post-race	48 h post-race	ANOVA results	
TC [mmol/l]	3.6–6.4	5.3 ± 0.3	ND	4.7 ± 0.7*	4.5 ± 0.5**	ND	4.4 ± 0.4**	**F**_**3,18**_ **=** **10.6**	***p*** **<** **10** ^**−3**^
LDL-C [mmol/l]	3.5	3.3 ± 0.3	ND	2.8 ± 0.5**	2.3 ± 0.5***	ND	2.5 ± 0.5**	**F**_**3,18**_ **=** **18.8**	***p*** **<** **10** ^**−3**^
HDL-C [mmol/l]	0.7–1.7	1.5 ± 0.3	ND	1.7 ± 0.3**	1.9 ± 0.3**	ND	1.5 ± 0.2	**F**_**3,18**_ **=** **24.4**	***p*** **<** **10** ^**−3**^
TG [mmol/l]	0.4–1.8	1.1 ± 0.5	ND	0.4 ± 0.1*	0.4 ± 0.2*	ND	0.9 ± 0.3	**F**_**3,18**_ **=** **9.61**	***p*** **<** **10** ^**−3**^
Fatty acids [mmol/l]	0.1–0.9	0.4 ± 0.1	2.3 ± 0.2***	1.4 ± 0.4**	1.09 ± 0.3*	0.6 ± 0.2	0.5 ± 0.2	**F**_**5,30**_ **=** **54.1**	***p*** **<** **10** ^**−3**^
βHB [mmol/l]^a^	0.03–0.30	0.10 ± 0.03	0.30 ± 0.11**	0.21 ± 0.05*	0.18 ± 0.08	0.08 ± 0.02	0.07 ± 0.02	**F**_**5,30**_ **=** **21.7**	***p*** **<** **10** ^**−3**^
Glycerol [μmol/l]^a^	28–108	38 ± 17	411 ± 142***	224 ± 105**	194 ± 138**	69 ± 22**	59 ± 17	**F**_**5,30**_ **=** **28.3**	***p*** **<** **10** ^**−3**^
TC/HDL-C	4.0	3.7 ± 0.5	ND	2.8 ± 0.3***	2.4 ± 0.3***	ND	3.1 ± 0.6**	**F**_**3,18**_ **=** **39.1**	***p*** **<** **10** ^**−3**^
LDL-C/HDL-C	4.5	2.3 ± 0.5	ND	1.7 ± 0.3***	1.3 ± 0.3***	ND	1.8 ± 0.6***	**F**_**3,18**_ **=** **39.2**	***p*** **<** **10** ^**−3**^
TG/HDL-C	3.5	0.8 ± 0.4	ND	0.2 ± 0.1*	0.3 ± 0.1*	ND	0.7 ± 0.3	**F**_**3,18**_ **=** **9.44**	***p*** **<** **10** ^**−3**^
Atherogenic index of plasma (AIP)^b^		−0.169 ± 0.220	ND	−0.661 ± 0.152***	−0.663 ± 0.245**	ND	−0.218 ± 0.205	**F**_**3,18**_ **=** **18.9**	***p*** **<** **10** ^**−3**^

Statistically significant ANOVA results are shown in boldtype

*βHB* β-hydroxybutyrate, *HDL-C* high -density-lipoprotein cholesterol, *LDL-C* low-density-lipoprotein cholesterol, *TC* total cholesterol, *TG* triglycerides, *ND* not determined

* *p* < 0.05, ** *p* < 0.01, *** *p* < 0.001 versus the respective pre-race value; the significance levels shown with asterisks refer to *p* values with the sequential Bonferroni–Holm correction

^a^The ANOVA results and significance levels pertain to log_10_-transformed data

^b^AIP = log_10_(TG/HDL-C) (Dobiášová and Frohlich 2001)

### Blood antioxidant status (see Table 7)

The blood activities of GPX, CAT, GR and SOD, and plasma TBARS level did not change significantly during the observation period. Mean GSH level was fairly stable during the run, but peaked transiently (+58 %) 24 h post-race. Mean plasma TAS increased but slightly (+26 %) after 12 h running and then normalized. During the run, TAS correlated moderately with actual running speed (*R* = 0.60, *p* < 0.001), WBC, neutrophil, and monocyte counts (*R* = 0.40, *R* = 0.45, and *R* = 0.45, respectively, *p* < 0.01 for all).

**Table 7:** Ultra-marathon-induced changes in blood antioxidant status

Variable	Reference range	Pre-race	12 h running	24 h running	48 h running	24 h post-race	48 h post-race	ANOVA results	
SOD [IU/g Hb]	1102–1601	1274 ± 341	974 ± 101	1021 ± 276	881 ± 135	943 ± 398	941 ± 237	**F**_**5,30**_ **=** **2.92**	***p*** **=** **0.029**
GPX [U/g Hb]	27.5–73.6	48.3 ± 17.0	44.2 ± 11.7	47.2 ± 11.7	47.8 ± 13.2	44.9 ± 11.2	48.5 ± 14.1	F_5,30_ = 0.24	*p* = 0.94
GR [U/g Hb]		27.1 ± 4.1	32.6 ± 6.5	40.9 ± 13.4	35.0 ± 9.6	39.3 ± 8.3	35.1 ± 8.7	F_5,30_ = 2.22	*p* = 0.078
CAT [k/g Hb]		225 ± 52	213 ± 64	232 ± 57	194 ± 43	191 ± 50	184 ± 30	F_5,30_ = 1.42	*p* = 0.24
Uric acid [mmol/l]	3.4–7.0	5.9 ± 1.0	6.8 ± 2.0	6.1 ± 1.8	5.1 ± 1.1	5.3 ± 1.0	4.8 ± 0.7	**F**_**5,30**_ **=** **4.21**	***p*** **=** **0.005**
GSH [μg/mg Hb]		3.40 ± 0.75	3.80 ± 0.47	3.70 ± 0.47	3.67 ± 0.48	5.80 ± 0.95**	4.91 ± 1.17	**F**_**5,30**_ **=** **11.22**	***p*** **<** **10** ^**−3**^
TAS [mmol Trolox/l]	1.30–1.77	1.28 ± 0.05	1.61 ± 0.17*	1.31 ± 0.10	1.43 ± 0.18	1.28 ± 0.16	1.35 ± 0.16	**F**_**5,30**_ **=** **6.39**	***p*** **<** **10** ^**−3**^
TBARS [nmol/ml]		7.03 ± 0.79	7.42 ± 0.88	7.63 ± 1.02	7.19 ± 1.36	5.59 ± 1.56	7.01 ± 2.12	F_5,30_ = 2.17	*p* = 0.11

Statistically significant ANOVA results are shown in boldtype

*CAT* catalase, *GSH* reduced glutathione, *GR* glutathione reductase, *GPX* glutathione peroxidase, *SOD* superoxide dismutase, *TAS* total antioxidant status, *TBARS* thiobarbituric acid-reactive substances

* *p* < 0.05, ** *p* < 0.01 versus the respective pre-race value; the significance levels shown with asterisks refer to *p* values with the sequential Bonferroni–Holm correction

## Discussion

This report is a time-expanded and recovery period-supplemented sequel of our earlier paper on acute metabolic responses to a 24 h ultra-marathon run (Waśkiewicz et al. 2012). Additionally, we have studied changes in two cardiovascular risk markers (serum hs-cTnNT and NTproBNP), Fe status and in several major components of the blood antioxidant defense. In contrast to other investigations, in both our studies we measured a variety of metabolic, oxidative stress, and cell damage indices also during the race. Importantly, the runners taking part in this study have begun their long-distance running careers fairly late in their lives and were not engaged earlier in any regular sports activities. While the group involved was much smaller than previously (7 vs. 14 participants, including 3 runners who took part in both events), the changes in physiological and metabolic indices during the first 24 h of the race closely followed those found in our earlier study, indicating reliability of the data.

### Metabolic responses to ultra-endurance exercise

The most characteristic metabolic response to ultra-endurance exercise is a distinct shift in blood lipid profile related to an increase in fat oxidation. We did not measure the latter in either a direct or indirect way in this study. However, the major elevations in serum FFA, glycerol and βHB during the run, mainly during the first 12 h, evidenced a substantial mobilization of systemic fat stores during muscular work (Helge et al. 2007) and an increased supply of substrate for intramuscular fat oxidation. This assumption finds additional support in the absence of significant changes in blood LA and glucose. An important regulatory role in the increased fat usage is played by the decreasing level of malonyl-CoA, the inhibitor of carnitine acyltransferase I (Winder et al. 1989). We also found a marked (−63 %) decline in mean serum TG during the race, which was associated with significant falls in serum TC and LDL-C levels, a significant elevation in serum HDL-C, and marked decreases in TC/HDL-C, LDL-C/HDL-C, and TG/HDL-C ratios. Most lipid profile parameters and ratios, except for LDL-C and TC, showed a considerable tendency for returning to pre-run values 48 h post race. Similar post-race changes in serum levels of TG, HDL-C, LDL-C, and TC were reported by Wu et al. (2004). Noteworthy, all the runners had a favorable lipid profile already at the start of the race, including a low (i.e. negative) value of AIP, which was reported to be inversely correlated with particle size of LDL and HDL (Dobiášová and Frohlich 2001). It may be presumed that this was, most likely, consequent on the high amount of weekly exercise (see Table 1), which is probably more important than exercise intensity for the beneficial effect of endurance exercise (see Kraus et al. 2002). Notably, basic lipid profile of our runners matched perfectly that reported in a group of much younger male athletes (mean age ± SEM 33.6 ± 1.1 years) recruited from triathlon (76 %), biathlon (12 %), running (8 %), and swimming (4 %) teams (Olchawa et al. 2004).

Prolonged strenuous muscle activity raises the need for carbohydrates, both for energy production in the working muscles and providing glucose for the brain. Since bodily carbohydrate stores are limited, an adequate intake of carbohydrates is considered crucial for endurance athletes (Zaryski and Smith 2005; Burke 2007). Lack of significant changes in blood glucose or LA levels in our runners during the race indicates that carbohydrate supply during the competition was sufficient. The increased exercise-related need for energy during the run could have been covered in part by enhanced hepatic βHB production.

### Muscle damage and inflammatory markers

The results of this study are in line with the common knowledge that prolonged endurance exercise causes increases in a number of established markers of muscle damage and the related inflammatory phenomena. The latter included large increases in serum activities of CK, LDH, AST, and ALT, which were apparent already at 12 h and continued until the end of the run. It is generally accepted that such increases result from enzyme leakage from skeletal muscles or other tissues due to mechanical damage or increased membrane permeability related to the emergence of exertional rhabdomyolysis. The fact that serum GGT activity did not increase throughout the race and recovery period implies that the primary source of all these enzymes was muscle, and not liver damage (Haralambie 1976; Bassini-Cameron et al. 2007; Banfi et al. 2012). A support for this presumption is also in the much higher (25-fold) relative increase in AST activity than that (sevenfold) in ALT activity, as the latter enzyme abounds in the liver rather than in muscles. Most of these activities showed a marked shift toward their respective pre-race values 24–48 h post-race, indicating a rapid progress of muscle healing.

The differences in the relative magnitudes of individual enzyme responses to exercise-induced stress may be related to dissimilarities in the sizes, shapes, and rates of penetration of these enzyme molecules through the sarcolemma, or in their respective routes from the interstitial fluid into circulation (via lymphatic system or direct entry from capillaries) (Havas et al. 1997). Similar differences in the dynamics of changes in serum activities of CK, LDH, AST, and ALT were found in marathon, ironman, and ultra-endurance runners (Suzuki et al. 2006; Bessa et al. 2008; Kim et al. 2009). There were considerable distinctions in the time courses of enzyme activity changes in relation to the phase of the respective run/recovery period (cf. Suzuki et al. 2006; Kim et al. 2009; and this study). These differences likely reflect the respective intensity characteristics of the various endurance events (e.g., a higher degree of walking/jogging during the second half of runs of longer duration) and time dependence of the phenomena underlying cell damage.

Acute inflammatory response in our runners was evidenced, inter alia, by increases in total numbers of circulating neutrophils and monocytes. This response to exercise-induced muscle damage typically involves early invasion of neutrophils that are mobilized by cytokine-mediated demargination from the lung vasculature or released from bone marrow in response to increased catecholamine levels (Tidball 2005; Butterfield et al. 2006). Our finding that peak values of WBC, neutrophil, and monocyte counts coincided with peak IL-6 level and that there was positive correlation between these indices for the entire observation period (*R* = 0.82, *R* = 0.84, and *R* = 0.67, respectively, *p* < 0.001 for all) is in line with earlier reports (Toumi et al. 2006; Wallberg et al. 2011). The main source of IL-6 during exercise are contracting muscles (Bruunsgaard et al. 1997; Pedersen 2009). IL-6 has long been considered an inflammatory cytokine, but it can also exert strong anti-inflammatory effect by boosting production of anti-inflammatory cytokines and inhibiting production of pro-inflammatory cytokines (Petersen and Pedersen 2006), and may act as a mediator of muscle healing (Toumi et al. 2006). Notably, its response to exercise is not preceded by an increase in TNFα and is not obligatorily related to muscle damage (Pedersen 2009), and exercise intensity but not duration is the key determinant of the response during endurance exercise of >12 h duration (Wallberg et al. 2011). Hence, it has been postulated that acute exercise-induced elevations in serum IL-6 are more important for metabolic than for immunological responses (Walsh et al. 2011) and its major role in working muscles is that of an energy sensor (Pedersen 2012). This postulate is consistent with our finding of considerably stronger (*p* = 0.04) correlation of serum IL-6 level with actual running speed than with total distance covered. Moreover, in this study serum IL-6 level correlated negatively with serum TG (*R* = −0.63, *p* < 0.001) and positively with serum FFA (*R* = 0.70, *p* < 0.001), glycerol (*R* = 0.73, *p* < 0.001) and βHB levels (*R* = 0.70, *p* < 0.001), supporting the postulated role of this myokine for the enhancement of lipid oxidation during muscular work (Pedersen 2009, 2012).

The course of changes in serum levels of muscle damage and inflammatory markers and a modest correlation between IL-6 and hsCRP (*R* = 0.39, *p* = 0.01) in our study is in line with the view that IL-6 induces liver CRP production (Fischer 2006; Devaraj et al. 2009). Still, serum hsCRP level correlated much stronger than serum IL-6 level with distance covered (*p* < 0.001), indicating that the key determinants of these indices differ, and hence these indices may reflect different muscular phenomena.

### Cardiac biomarkers

Regular physical activity is considered beneficial for cardiovascular health. However, prolonged strenuous exercise may be associated with adverse cardiovascular consequences in athletes (see “Introduction”) and abnormal release of cardiac biomarkers (Trivax et al. 2010; Le Goff et al. 2012). The preferred markers of cardiac dysfunction are cardiac troponins T and I, NTproBNP, and CK and its MB isoform (for review see Banfi et al. 2012).

In the present study, the highest increases in hs-cTnT were recorded after 12 h running, while NTproBNP reached the highest levels at mid-race. Notably, individual serum NTproBNP levels exceeded the suggested decision cut-point for the detection of heart failure (125 pg/ml; Cowie et al. 2003) at some time points during the race in all runners and remained abnormally high in most of them at the end of the run, but normalized 48 h post-race. In contrast, serum hs-cTnT level crossed shortly its respective URL (0.100 μg/L; Mingels et al. 2009) only in two competitors after 12 h running, and in one runner at a later time point. The associations found between actual running speed and serum hs-cTnT or NTproBNP level (see the “Results” section), and between serum NTproBNP level and run duration (*R* = 0.55, *p* < 0.01) may imply that exercise intensity and duration were both responsible for elevated release of the cardiac biomarkers. These results support the suggestion (Legaz-Arrese et al. 2011) that prolonged strenuous exercise-related release of these markers does not reflect a clinically threatening heart injury, but are due—in case of NTproBNP - to temporary high myocardial wall stress (Cowie et al. 2003) or—in case of hs-cTnT—to transient and reversible increases in myocardial sarcolemma permeability with no enduring structural damage (Shave et al. 2010). The latter ‘leakage’ could be triggered by increased production of oxygen-free radicals and oxidative damage (Nie et al. 2010). One should realize that the apparent lack of evidently unfavorable, permanent effect(s) of the ultra-endurance effort in our study may not reflect the actual risk from such activity in general population. This is because the participating runners have been engaged in ultra-endurance running for a considerable time (see Table 1) and hence they represent a particularly fit subgroup of those who attempt to start their endeavors with this type of physical activity, as those who experienced some negative effects of this activity (or have had less stamina) likely dropped out.

### Acid–base balance and electrolytes

No substantial hyponatremia was observed in our runners during the observation period, implying appropriate hydration. The hypocapnic respiratory alkalosis that developed in our runners was consistent with earlier reports (Hanson et al. 1982; Waśkiewicz et al. 2012). Since this ultra-marathon race was held at temperatures well below core body temperature, a contribution of hyperthermia to this effect (Abbiss et al. 2007) seems negligible. The most likely cause of the observed progressive decline in capillary blood ionized calcium seems alkalosis, conducive to increased binding of calcium to albumin (Baird 2011). This presumption is supported by a significant correlation between [Ca^2+^] and pH (*R* = −0.57, *p* < 0.001). Significant (*p* < 0.01) associations between serum IL-6 and pCO_2_ (*R* = −0.45), pH (*R* = 0.48) and (pCO_2_/HCO_3_^−^) ratio (*R* = −0.50), which are consistent with our earlier study (Waśkiewicz et al. 2012), may imply that hyperventilatory alkalosis could modulate inflammatory response by facilitating IL-6 release, as such effect of hypocapnia has been found in endotoxin-stimulated human whole blood cell cultures (Kimura et al. 2008).

Consistent with earlier reports (Lindinger 1995; Sahlin and Broberg 1989) was a progressive rise in blood [K^+^] during the run. This effect, which is due to a continuing net loss of K^+^ from contracting skeletal muscle cells during a prolonged exercise, is presumably caused not by increased cell membrane permeability, but rather stems from active transport of K^+^ ions by the ATP-dependent Na^+^/K^+^ pump across the membrane.

### Iron status

One of the important issues facing endurance athletes is potential Fe deficiency. The most common laboratory measure of Fe status is serum ferritin, but given the acute-phase reactant properties of the latter (Witte 1991), the sTfR level is considered a superior index of functional Fe deficiency (Mast et al. 1998; Malope et al. 2001) and of an enhanced erythropoiesis. Notably, sTfR level is elevated in Fe deficiency anemia, but not in the anemia of inflammation, although it may decrease transiently during acute inflammation (Beguin 2003). In the present study, a sustained 48 h run led to a progressive rise in serum ferritin level that correlated negatively with serum Fe level and, as suggested by strong correlations with muscle damage markers (CK, AST, LDH, and ALT) and hsCRP (see the “Results” section), was likely related to exercise-induced inflammation. The observed decline in sTfR levels during the race might have been stimulated by exercise-induced acute inflammation, as suggested by Beguin (2003). Notably, serum sTfR remained within normal range in all runners during the consecutive time points of the race, but it dropped slightly below the lower reference limit 24 h post-race in three competitors, in whom Hb and Hct were also the lowest. Moreover, the absolute levels of sTfR and sTfR/logFer index remained within the respective normal reference ranges (Suominen et al. 1998). Progressive increases in sTfR and sTfR/logFer index are considered indicative of increased risk of Fe deficiency (Suominen et al. 1998). Thus, the prevalence of normal levels of sTfR and sTfR/logFer index, as well as a progressive declines in both these parameters, most likely related to exercise-induced inflammation, may imply that neither the Fe storage pool nor erythropoiesis in our runners were substantially affected.

### Blood antioxidant status

It is well known that heavy physical exercise enhances oxygen consumption and production of reactive oxygen species (Finaud et al. 2006; Mastaloudis et al. 2001; Westerblad and Allen 2011). Sources of these species during contractile activity include electron leakage from respiratory complexes I and III, the phagocytic activity of WBC mobilized due to muscle fiber injury, and activities of phospholipase A_2_, xanthine oxidase, and NADPH oxidase (Knez et al. 2006; Powers et al. 2011). Our study showed that activities of antioxidant enzymes were not considerably affected by the exercise stress. This might have been related to relatively low intensity of the exercise. Plasma content of uric acid, a product of purine nucleotides degradation that functions as an antioxidant (Ames et al. 1981), increased mostly over the first 12 h of the race, when a relatively high exercise intensity and a high rate of energy consumption should have triggered activation of adenylate kinase and AMP signaling networks to ensure cellular energy homeostasis (Dzeja and Terzic, 2009). This observation is in line with earlier findings (Mastaloudis et al. 2001) and supports the hypothesis on enhanced purine oxidation and xanthine oxidase-catalyzed uric acid formation during exercise (Sjödin et al. 1990). Blood GSH level, expressed per mg of Hb, showed no change for the duration of the race, but rose significantly post-run. As there was no considerable increase in antioxidant enzymes activity during the observation period, the most likely reason for the increase in GSH was the tendency toward lower levels of Hb, RBC, and Hct evidenced during the race, and in particular during the recovery. Considering that almost all blood GSH is contained in the erythrocytes (Sen and Packer 2000), this hypothesis is additionally supported by our finding of moderate negative correlations between blood GSH and Hb (*R* = −0.49, *p* < 0.001), RBC count (*R* = −0.51, *p* < 0.001), and Hct (*R* = −0.48, *p* < 0.01).

The hypothesis that ultra-endurance exercise increases oxidative stress was supported by the observation of changes in plasma TBARS, a marker of lipid peroxidation, and in plasma TAS, a measure of blood total antioxidant capacity. The highest plasma TBARS level was found mid-race, while it returned to baseline after 48 h of recovery. Noteworthy, both pre-, mid-, and post-race plasma concentrations of TBARS were comparable to those reported in half and full ironman triathletes (Knez et al. 2007), but markedly higher than those reported in our previous studies in professional soccer players (Kłapcińska et al. 2005) and ultra-marathoners (Waśkiewicz et al. 2010). These discrepancies could be due to higher physical loads to which the runners were subjected shortly prior to the ultra-marathon. Regarding TAS of plasma, it attained the highest level (*p* < 0.05) after 12 h running, i.e. during the race phase characterized by a maximum running speed, then remained at fairly stable baseline levels until the finish of the race and over the recovery. This effect may simply indicate an initial response to oxidative stress. Taking into account that uric acid is the main determinant of TAS (reviewed in Bartosz 2003), increases in TAS may be associated with parallel increases in plasma urate that also peaked after 12 h running, although the association between TAS and serum uric acid content did not reach significance. These findings suggest a full compensation of the increased oxidative stress by antioxidative defenses system in these runners, which may be due to training-related adaptive changes. This observation finds support in the marked while transient elevation in blood GSH in relation to blood Hb (+58 % vs. the respective value at the completion of the race, *p* = 0.0012 by the Student *t* test for dependent variables) after the 1 day of recovery; this likely resulted mostly from reduction of glutathione disulfide accumulated during the run by GR, as the simultaneous decrease in Hb (−13 %) could only moderately contribute to this change.

### Concluding remarks

In summary, the 48 h ultra-marathon-related strain caused severe muscle, but not liver, damage and induced an acute inflammatory response. These phenomena were evidenced by abnormally high serum levels of muscle enzymes, marked leukocytosis, several fold rise in IL-6 and hsCRP, and marked increases in blood levels of cardiac stress biomarkers (NTproBNP and hs-cTnT), but no sizeable shift in the blood pro-oxidant/antioxidant balance. The time course of changes in these characteristics, and particularly the marked shifts toward pre-race values during the 48 h of post-race recovery indicate that the changes observed during the run were mechanistic consequences of strenuous exercise and did not herald a lasting myocardial injury or any other pathology. The predominant ventilatory response to a sustained 48 h running was a hypocapnic respiratory alkalosis maintained until the end of the race and associated with moderate hyperkalemia and hypocalcemia, but not hyponatremia. There is some circumstantial evidence to assume that hyperventilatory hypocapnia could modulate inflammatory response by stimulating or facilitating IL-6 release. The relative stability of RBC—related indices throughout the race, which was associated with moderate declines in blood sTfR level and sTfR/log_10_Fer index, implies that neither the iron pool nor erythropoiesis were substantially affected during the ultra-endurance event. These findings seem to imply that starting high physical activity at middle age does not increase health risk in well-conditioned middle-aged men.
